# Using Item Response Theory Models in Scaling Severity Scores in Alcohol Research—A Tutorial

**DOI:** 10.1111/dar.70053

**Published:** 2025-10-26

**Authors:** Daniel Schulze, Kim Bloomfield, Pimrapat Gebert, Ulrike Grittner

**Affiliations:** ^1^ Institute of Biometry and Clinical Epidemiology Charité Universitaetsmedizin Berlin Berlin Germany; ^2^ Centre for Alcohol and Drug Research Aarhus University Copenhagen Denmark; ^3^ Department of Psychology University of Johannesburg Johannesburg South Africa

**Keywords:** alcohol harms due to others drinking behaviour, Denmark, item response theory

## Abstract

**Introduction:**

Questionnaires are frequently used in the assessment of drug and alcohol dependence.

**Methods:**

Typically, sum scores are used for aggregation, which come with several underlying assumptions. If untested psychometrically, the reliability and sufficiency of a sum score as a measure of the characteristic of interest remain unclear. A simple sum score, for example, does not weight the items and therefore each item contributes with the same level of importance to the total. However, weighting more severe symptoms higher would often be desirable. Additionally, sum scores do not reflect the unreliability of the questionnaire items; i.e., the proneness of an item to measurement error. Unreliability reduces the effects of interest, e.g., an analysis of group differences on a score will underestimate true effects. Latent variable models account for measurement error and provide reliable estimates of a person's score on the underlying constructs.

**Results:**

In this paper, we showcase the application of item response theory models from psychometrics for the analysis of categorical questionnaire data. Using non‐technical language, we introduce the most common models and discuss model parameters, model fit, and group comparisons. These aspects allow for the evaluation of the assumptions underlying a sum score.

**Discussion and Conclusions:**

We introduce these concepts with the help of real data from the Danish alcohol and drug consumption survey from 2011, using items on alcohol harms to others. The data and the full reproducible code in STATA and R are provided. Reporting guidelines are given as well as a discussion of pitfalls and limitations.

**Summary**

- Item response theory methods are useful for the scaling of scores that are based on items such as alcohol harms to others severity scores.
- Scores scaled by item response theory are more reliable than simple summary scores.
- In this paper we present an easy‐to‐follow introduction on how to apply item response theory methods to scale scores.
- We provide R and Stata Codes with explanations and examples.
- The example data set comes from the 2011 Danish national alcohol and drug survey.

## 1 Introduction

Alcohol and drug research frequently rely on standardised questionnaires to assess substance use severity, with instruments like the Alcohol Use Disorders Identification Test (AUDIT) being widely utilised [1, 2]. These tools are typically designed for brevity and ease of administration, quantifying the severity of substance dependence by a single sum score. However, sum scores can be oversimplifying and thus hamper the measurement of severity [3]. In this paper, we critically examine the assumptions underlying sum scores and introduce item response theory (IRT) as an analytical framework for alcohol and drug researchers, offering a way to test and relax these assumptions. When calculating a sum score of a questionnaire, several implicit assumptions are made. Firstly, it is assumed that all items reflect a single latent trait (e.g., alcohol dependence). A latent trait is a subject's feature that cannot be assessed directly but must be inferred from indicators that themselves are caused by the latent trait. Addiction severity is an example of a latent variable. Secondly, as all items contribute with equal weight to the sum score, it is assumed that all items reflect the latent trait with the same quality and intensity. For example, in the AUDIT score, the possible answer: ‘I drink alcohol two to four times a month’ (consumption) and the response ‘Once a month I fail to do what is expected from me because of my drinking’ (symptom) have the same score of two points each. The assumption that these two questions are equally relevant and indicative for alcohol dependence seems implausible. However, this very assumption is made when calculating a sum score. Thirdly, measurement error is assumed to be absent. Measurement error, in a technical sense, is the deviation in the observed answer of a respondent compared to the ‘true’ answer that corresponds to the true (unmeasurable) value. Such errors might be due to misinterpretation of the question or its answer categories, or due to responding to the question in a socially desirable manner. Any measurement error is incorporated into the final sum score, artificially increasing its variance and, in turn, making it difficult to detect truly existing associations and group differences. These three implicit assumptions usually remain untested and their impact on results unquantified. It thus stands to reason that these assumptions are often violated and study results based on sum scores might be biased.

IRT originated in psychological testing and describes the probabilistic relationship of categorical items with an assumed underlying latent variable that cannot be measured directly [4]. Various models belong to the IRT framework [5, 6], characterised by the number of response options in the single items and the number of assumptions about the underlying score. Detailed presentation of psychometrical theory and applied analysis can be found in a textbook by Price [7]; for those who prefer less volume, an applied introduction to questionnaire development and validation is given in a comprehensive paper by Kyriazos and Stalikas [8]. After introducing a data example, we will go through prominent IRT models with increasing mathematical complexity (i.e., with relaxing assumptions).

## 2 Methods

### 2.1 Data

To illustrate IRT models, we used survey data of the Danish alcohol and drug consumption survey 2011 [9]. We chose an 8‐item scale of alcohol harms due to others' drinking behaviour (AHTO). The questions are shown in Table 1 and were introduced by the sentences ‘The next questions deal with various problems that one can experience due to another's drinking. Think back over the last 12 months, has it happened that a drunken person has …’. Response categories involved the three statements *yes, more than two times*; *yes, once or twice*, and *no*. These questions were answered by a total of *N* = 5133 participants. For the sake of simplicity, the analysis was restricted to 5101 participants with no missing values in the relevant variables. Details of the study design are described elsewhere [10].

**TABLE 1:** Results for items of the alcohol harms due to others' drinking behaviour scale for the Rasch, partial credit (PC), and 2Pl model (*n* = 5101 respondents) (*β*: Item difficulty, *α*: Item discrimination coefficient).

#	Item	Rasch	PC		2PL	
		*β*	*β*1	*β*2	*β*	*α*
1	Been kept awake at night due to noise from drunken persons	1.71	2.46	4.29	−1.27	0.84
2	Harassed you on the street or in a public place	1.73	2.57	4.39	−2.91	3.74
3	Yelled at you or insulted you in other ways	2.12	2.92	5.07	−2.79	2.77
4	Has made you afraid on the street or other public place	2.75	3.48	6.18	−2.52	1.52
5	Has insulted you at a party or other private event	2.85	3.73	6.49	−2.91	1.88
6	Ruined clothes or belongings	4.31	3.84	8.02	−4.66	2.10
7	Has physically harmed you	5.10	3.98	8.92	−5.68	2.23
8	Was responsible for an auto accident in which you were involved	6.50	4.06	10.40	−5.79	1.27

*Note:* The questions were introduced by the sentences ‘The next questions deal with various problems that one can experience due to another's drinking. Think back over the last 12 months; has it happened that a drunken person has …’.

### 2.2 Key Terms

#### 2.2.1 Latent Trait (*θ*)

A latent trait is the characteristic of the respondent or patient that a researcher or physician wants to assess. It cannot be measured directly but by a battery of single items. The extent of the latent trait (e.g., extent of suffering from AHTO) influences the probability of a respondent answering positively to a given item (e.g., if he or she experienced a specific harm).

#### 2.2.2 Item Characteristic Curve (ICC)

The ICC models the probability that a respondent endorses a specific item which is dependent on the respondent's latent trait (*θ*) and the item difficulty (*β*). For the Rasch model the ICC is given in formula nr. (1).

#### 2.2.3 Difficulty Parameter (*β*)

The difficulty (*β*) is a characteristic of an item. Each item has its own quantitative difficulty parameter representing how easy or difficult an item is for the respondent. Since the individual latent trait characteristic (*θ*) of the respondent and the item difficulty are measured on the same scale, there is a direct link between them. The difficulty (*β*) for item i is the ability level (*θ*) for those individuals who have a probability of 50% to endorse this item. Note that in our example on AHTO the difficulty of an item is to be interpreted as the severity of this item. In other words, endorsing a more severe item indicates a higher level of AHTO impact.

## 3 Results

### 3.1 Questions With Two Response Options: The Rasch Model

**FIGURE 1:**
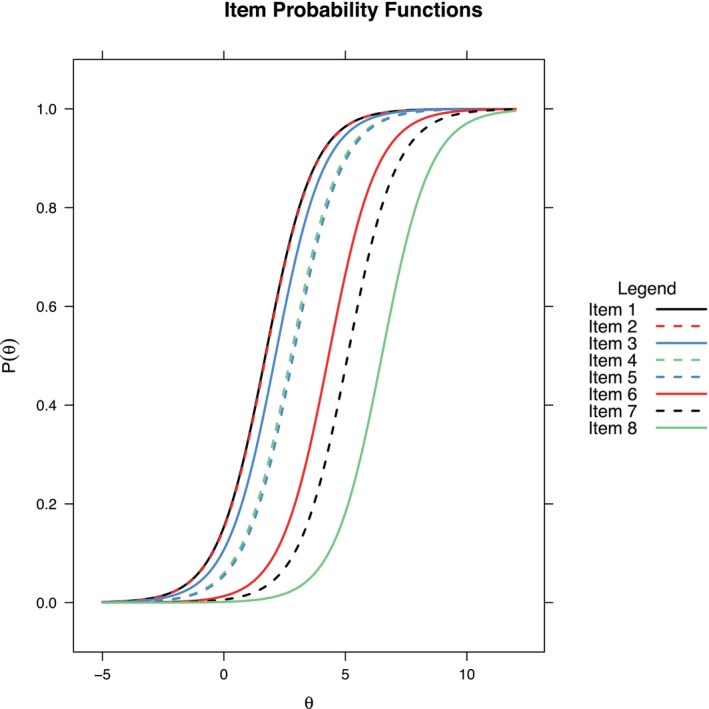
Item characteristic curves under the Rasch model, *x* axis: Latent trait *θ* (level of alcohol harms due to others' drinking behaviour severity), *y*‐axis: Probability of endorsing the specific item (8 items, *n* = 5133 respondents).

The mathematically simplest IRT analysis model, the Rasch model [5] corresponds to the sum score, but explicates the underlying assumptions of sum scores and allows testing them. To that end, the Rasch model contains two parameters: the item difficulty (i.e., responding positively to a dichotomous item) and the person's score on the latent variable. Because the latent variable cannot be observed directly, it lacks a natural metric, thus needing the model to establish one. Both parameters have ranges on the same abstract scale, called a logit scale due to the logarithmic relationship between responses and the latent variable (see Figure 1 for a representation). Mathematically, the Rasch model is expressed by the

(1) $$P\left(X_{\mathrm{pi}}=1|\theta ,\beta \right)=\frac{e^{\theta_{p}-\beta_{i}}}{1+e^{\theta_{p}-\beta_{i}}}$$

equation provided in formula number (1), with *X*_*pi*_ denoting the item response of person *p* on item *i* (0 for ‘no’ and 1 for ‘yes’), *θ*_*p*_ the latent person score, which is assumed to follow a normal distribution across all individuals with mean zero and standard deviation *σ*, meaning that zero represents the average person score across all included respondents. *β*_*i*_ is the item difficulty/severity of the respective item *i*. The item difficulties/severities range on the same scale as does the person score. An item with difficulty/severity zero would thus be an item experienced by half of the respondents in the sample, thus being a typical or average person's experience.

We will explain the properties of the Rasch model with the help of the AHTO scale. For illustration, we have collapsed the two yes categories from the three possible responses for each item (*yes, more than two times*; *yes, once or twice*; and *no*) into one, resulting in binary responses.

IRT models in the statistical software R can be run with the mirt package (viable alternatives are ltm and TAM). After restricting a data frame to the items of interest, the mirt command estimates a Rasch model, and the mirt command returns the estimated parameters *β*_*i*_ (item difficulties/severities) per item and the variance *σ* for the individual characteristic *θ*. For brevity, the item parameters *β*_*i*_ are shown in Table 1, column 1 (Rasch model coefficients). The simplified R output in the supplement provides additionally the mean and variance of the latent variable: zero and 3.27 (resulting in *σ* = 1.81). The estimated score for each person can be drawn from the fscores() command from the model (R/Stata code in supplement).

All item difficulties are above 0, meaning that all items are affirmed by less than 50% of the respondents in this sample (see Table 1, column 1, Rasch model coefficients). The least difficult item was ‘*Been kept awake at night due to noise from drunken persons*’ with *β* = 1.71. The item difficulty/severity translates into a probability to endorse the item, which for the average person is *e* ^0–1.71^/(1 + *e* ^0–1.71^) = 15%. In the same manner, response probabilities can be calculated for any person score. A subject with a comparatively elevated AHTO score, e.g., one standard deviation above the mean, would thus have a probability of *e* ^1.81–1.71^/(1 + *e* ^1.81–1.71^) = 52% to endorse the item on being kept awake. An important property of IRT models is: If the item difficulty and the person's score are identical, the probability of responding ‘yes’ to an item of this person is 50%.

Another useful feature of IRT models can be illustrated with the most difficult item (‘*A drunken person was responsible for an auto accident in which you were involved*’). The item has a probability of endorsement of only 0.15% for a respondent with an average person score. That means even items that are scarcely endorsed can be modelled and have a probability greater than zero. Of course, this only holds as long as there is at least one respondent in the sample who endorsed the item.

Another important feature of the Rasch model is the fact that the ranking and differences of the item difficulties are independent of the actual sample that is used to estimate the item parameters. This characteristic of the Rasch model is a consequence of its model assumptions (uni‐dimensionality: (i) all items measure one latent trait; (ii) items are equally discriminating between respondents on different levels of the latent trait; (iii) the probability of a respondent endorsing an item is a function of the latent trait of the respondent and the item difficulty). With a different sample, we would thus draw the same conclusions about which item is the hardest to affirm and how much harder it is than the second‐hardest item. This property is sometimes called sample independence or specific objectivity. Note that we would *not* find the same numerical values for the *β*s in another sample. The item difficulties would instead be shifted by a simple additive constant that reflects the difference in the sample's average person score; that is, if the second sample would generally have higher AHTO levels, item difficulties are shifted to lower values, implying seemingly easier/less severe items. The item ranking would nevertheless be the same.

Our example comprises a large sample; however, such numbers are no prerequisite for the estimation of an IRT model. A general rule of thumb suggests at least 10 cases per item; i.e., 100 respondents for a 10‐item scale. This rule of thumb is simplistic because several factors impact the precision of latent variable models. MacCallum and Widaman [11] report the reliability and the correct structure (meaning that the data structure fits the model assumptions, i.e., uni‐dimensionality as the typical case) of the data as influential factors. Reliable items in a correctly set up structure require fewer subjects (as low as 60) than items with lower reliability measures or data with a structure that fits not ideally with the model assumptions (e.g., unidimensional, although the items are more or less split into two scales in actuality).

### 3.2 Testing Assumptions and Model Fit

Achieving sample‐independent measurement, wherein person ability estimates remain invariant across different item subsets and item difficulty calibrations hold across diverse person samples, represents a psychometric ideal. The Rasch model offers a pathway to this ideal, predicated on stringent assumptions. Specifically, the model mandates unidimensionality—that a single latent trait accounts for item responses—and that all items demonstrate equivalent discrimination, thereby reflecting the latent variable with equal fidelity (differing only in difficulty). When these conditions are met, the resulting raw sum scores serve as sufficient statistics for the person parameters. Consequently, the readily interpretable sum score can be confidently employed as a direct proxy for the model‐derived latent trait estimate, simplifying interpretation without sacrificing fundamental measurement properties. Whether these rigorous prerequisites are fulfilled can be appraised by assessing model fit indices. Even though there are various item‐level statistics available, for the purpose of model comparison, we will concentrate on global model fit. First, there is a statistical test on model fit which compares the data structure to the proposed model [12]. Differences between the assumed model and the data will yield a small *p*‐value. Our example returns a *p* < 0.01, indicating that the Rasch model does not fit the data very well (see Table 2). However, significance tests strongly depend on sample size; i.e., with large samples, they reveal even small differences as significant. Therefore, model fit should be assessed with a focus on fit indices that express the degree of misfit independently from sample size (such as an effect size). Common fit indices include: the root mean square error of approximation (RMSEA), the standardised root mean square residual (SRMSR), the Tucker‐Lewis index (TLI) and the comparative fit index (CFI). These indices range from zero to one, where zero indicates perfect fit for RMSEA and SRMSR and one indicates perfect fit for TLI and CFI. Values below 0.05 for RMSEA and SRMSR [13] and values above 0.95 for TLI and CFI provide good model fit [14].

**TABLE 2:** Model fits for Rasch, polytomous Rasch, and 2Pl models (8 items, *n* = 5101 respondents).

	M2	df	*p*	RMSEA (95% CI)	SRMSR	TLI	CFI
Rasch	689.70	27	< 0.01	0.07 (0.06–0.07)	0.08	0.90	0.91
PR	196.90	19	< 0.01	0.04 (0.04–0.05)	0.09	0.96	0.96
2PL	126.67	20	< 0.01	0.03 (0.03–0.04)	0.03	0.98	0.99

Abbreviations: CFI, comparative fit index; CI, confidence interval; PR, polytomous Rasch model; RMSEA, root mean square error of approximation; SRMSR, standardised root mean square residual; TLI, Tucker‐Lewis index.

All statistics denote insufficient fit for the Rasch model (see Table 2). Note that the RMSEA is the only fit index that is accompanied by a 95% confidence interval (CI), giving insight into the precision of the RMSEA. Due to the large sample, the CI is narrow. It does not include the cut‐off of 0.05, indicating inadequate model fit. Given the present data, we thus need to apply different models. Our conclusion is that the Rasch model for binary items does not provide adequate fit.

### 3.3 More Than Two Response Categories: Polytomous Rasch Model

The polytomous Rasch model [15] is an extension of the Rasch model for more than two answer categories and is sometimes also called the partial credit model. The necessary extension is straightforward: instead of a single item difficulty/severity, multiple thresholds are included to model the ‘difficulty/severity’ in moving from one ordinal response category to the next. The partial credit model is expressed by formula (2),

(2) $$P\left(X_{\mathrm{pi}}=j|\theta ,\beta \right)=\frac{e^{\sum_{k=0}^{j}\left(\theta_{p}-\beta_{\mathrm{ik}}\right)}}{\sum_{h=0}^{c-1}e^{\sum_{k=0}^{h}\left(\theta_{p}-\beta_{\mathrm{ik}}\right)}}$$

where there are *c* response categories (*c* > 2), which are modelled with *c* − 1 thresholds; i.e., we now have multiple *β*s per item. In formula (2) *X*_pi_ is the item response of person *p* on item *i* (with answer category *j* ≤ *c*), *θ*_p_ is the latent person score, *β*_*ik*_ is the item difficulty/severity of the respective item in category *k* (*k* ≤ *c*). Mathematically this is done by summing across all response categories up until the category that was actually chosen by a participant *p* for item *i*.

As the original response scale of the AHTO questionnaire involved three categories (*yes, more than two times*; *yes, once or twice*; and *no*), the PC model is a natural choice. In mirt, a PC model is estimated simply by using the data with its original three ordinal response categories (R/Stata code in supplement). Table 1 (column 2, PC model coefficients) shows the two difficulty parameters per item: *β*_1_ for crossing over from *no* to *yes once or twice* and *β*_2_ for switching from the latter to *yes, more than two times*. Naturally, *β*_2_ is larger than *β*_1_ in every case, indicating that a higher AHTO score is needed for endorsing *yes once or twice*. This result validates the intuitive order of response categories. However, the PC model can also yield parameters that conflict with the initially assumed order, indicating problems with the respective item.

With respect to model fit, the polytomous Rasch model fares better than its binary counterpart (see Table 2). Except for the SRMSR, all fit indices indicate proper model fit. The M2 statistic has decreased considerably, suggesting an increase in model fit.

### 3.4 Weighing Questions Differently: 2PL Model

The Rasch model assumes that all binary items reflect the latent variable equally well. This assumption can be relaxed in cases where good model fit cannot be achieved with Rasch‐type models. A disadvantage of relaxing this assumption is that the sum score of the scaled items is no longer an equivalent of the IRT person score and that sample independence is lost. However, in many practical applications, items do not conform to the stringent assumptions of the Rasch model, leading to the widespread adoption of two‐parameter logistic (2PL) models.^1^

The 2PL model [16] for dichotomous responses can be described by:

(3) $$P\left(X_{\mathrm{pi}}=1|\theta ,\beta ,\alpha \right)=\frac{e^{\alpha_{i}\cdot \theta_{p}-\beta_{i}}}{1+e^{\alpha_{i}\cdot \theta_{p}-\beta_{i}}}$$

**FIGURE 2:**
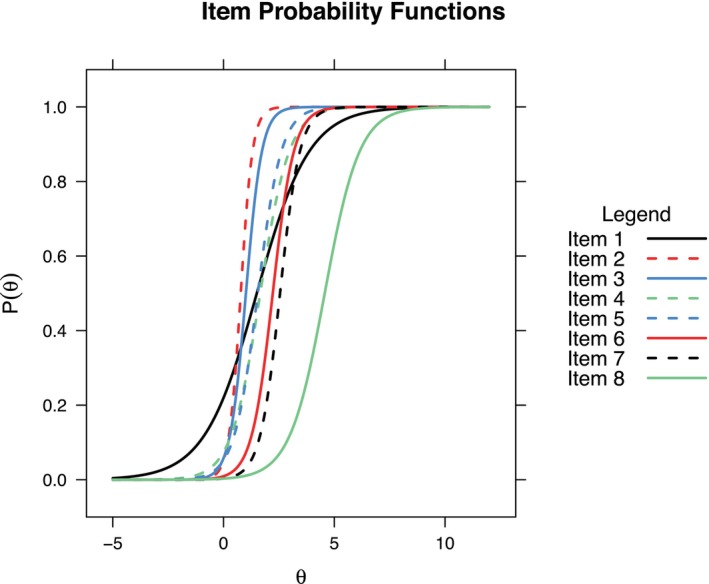
Item characteristic curves under the 2 PL model, *x* axis: Latent trait *θ* (level of alcohol harms due to others' drinking behaviour severity), *y*‐axis: Probability of endorsing the specific item (8 items, *n* = 5133 respondents).

In formula (3) *X*_*pi*_ is the item response of person *p* on item *i* (with 2 answer categories as in formula (1)), *θ*_p_ is the latent person score, *β*_*i*_ is the item difficulty/severity of the respective item. The discrimination parameter *α*_*i*_ (also called item loading) is now added to the model (1). A higher item discrimination corresponds to a better ability to discriminate between persons with different latent scores. This relates to the slope of the logarithmic curve, which becomes steeper for higher values of *α* (Figure 2). As the slope is not contained in Rasch‐type models, it equals one for all items (see parallel item curves in Figure 1).

Applying a 2PL model in mirt requires only changes to the itemtype argument^2^ (R/Stata code in supplement). For the dichotomous AHTO data, the item discrimination parameters differ substantially and range from 0.84 to 3.74 (see Table 1, column 3, 2PL model coefficients). An affirmative response to the item with the highest discrimination (*Has a drunken person harassed you on the street or in a public place?*) is more informative with regard to the person's AHTO score than the items with low discrimination (*Have you been kept awake at night due to noise from drunken persons?*). Note that the item difficulties have changed, although only slightly. This change is a consequence of the relaxed assumptions of the 2PL model. Model fit has increased further (see Table 2): All model fit indices display good fit. Note that the Two‐Parameter Logistic (2PL) model offers greater adaptability in analyzing test data compared to the stricter Rasch model. Because the 2PL model allows test items to vary not only in their difficulty but also in how well they differentiate between individuals of different ability levels (their ‘discrimination’), it can often provide a more accurate representation of how people are affected e.g., by alcohol consumption of others. However, this flexibility comes with a drawback: the simple total score (the sum of correct answers) becomes less straightforward to interpret. Since items can contribute differently to the overall severity estimate in a 2PL model, the sum score is no longer a direct or easily understood measure of a person's underlying AHTO affection, unlike in the Rasch model where, if its assumptions hold, the sum score has a clearer meaning (Figure 2).

### 3.5 Testing Comparability Between Groups: Measurement Invariance

Research questions frequently involve the comparison of different groups or the same group at different time points. In the following, we showcase group comparisons by the AHTO of young adults (19–28 years, *n* = 785) versus adolescents (15–18 years, *n* = 291). Valid group comparisons using IRT models require that test items function consistently across different groups. This means that item parameters—such as difficulty and discrimination—should be the same for all groups being compared. When items behave differently across groups, any observed differences in test scores may reflect problems with the measurement instrument rather than true differences in the underlying trait. This measurement bias can lead to incorrect conclusions about group differences, as the observed score gaps may be artefacts of how the items function rather than genuine differences in ability or the characteristic being measured. In this case, differences in the difficulty/severity of some items between two groups will translate into apparent group mean differences. The consistency of the relationships between items and the underlying latent trait across multiple groups is called *measurement invariance* (MI), whilst the opposite; i.e., items having different properties, is called *differential item functioning* (DIF). MI/DIF can be assessed by simultaneously estimating a model in several groups. Note that assessing group differences on sum scores just as well makes implicit assumptions about the underlying psychometric model. Sum score comparisons similarly will yield biased group differences if item parameters are not equal across groups; however, the researcher is utterly blind to the implicit models and assumptions.

We hypothesised that experiences of AHTO might differ in young adults and adolescents as their social environments are subject to significant changes in this period: legal restrictions on alcohol purchase change (at the age of 18, every type of alcohol can be bought legally in Denmark; however, beer and wine below an alcohol content of 16.5% can be bought at the age of 16). Additionally, most persons move out of their parent's home at some point and thus residence also differs between these age groups. However, before we can test group differences, we have to evaluate if the items function similarly in both age groups, meaning that MI is sufficiently given in our model. The prerequisites of comparing group means require that an IRT model possesses MI in three respects: (i) being uni‐dimensional in both groups; (ii) having the same item discrimination; and (iii) difficulty/severity parameters in both groups. These aspects can be tested by comparing model fit with different levels of restrictions, meaning including mathematical restrictions in the multigroup model, e.g., by equalising item discriminations across groups. MI/DIF analysis is thus initiated by estimating three subsequent multigroup models:

1. configural MI: testing uni‐dimensionality in both groups without further restrictions,
2. weak MI: additionally, equalising item discriminations across groups,
3. strong MI: additionally, equalising item difficulties/severity levels across groups.

Across these stages, the model becomes more restrictive, and a more restrictive model will have worse model fit if deviations of the data from the model are present. In R, the estimation of the three MI models is achieved via the multipleGroup function that extends the mirt command. Model restrictions are included with the invariance statement^3^ (R/Stata code in supplement). Models can then be compared step by step with the analysis of variance function in R. This gives a significance test for differences in model fit (∆*M*2) and differences in the fit indices. The latter should not exceed a decrease in CFI > 0.01 nor an increase in RMSEA > 0.015 [17]. If the test yields a significant result *and* the differences in fit indices supersede these cut‐offs, the assumptions of the more restrictive MI model are too strict, and it must be rejected.

We start by comparing the configural MI model with no further restrictions with the weak MI model assuming the same item discrimination values in both groups. We find a non‐substantial decrease in model fit for weak MI compared to configural MI (Table 3). Additionally, in the weak MI model, we used only 7 parameters compared to 40 in the configural MI model and achieved almost similar model fit values. Therefore, we move on to testing strong MI; maybe an even stricter model (additionally assuming equal item difficulty/severity parameters) across groups might hold. Here, model fit decreases substantially compared to weak MI, and the difference in CFI is clearly above the cut‐off, whilst the RMSEA does not increase substantially. Thus, weak MI can be retained, but strong MI is rejected. As such, the coefficients of the weak model can be interpreted validly (see Table 4), but not those of the strong model. For comparison, we added the model fit of a MI Rasch model as, i.e., a model mimicking the assumptions of a sum score (Table 3, last line). For the sum score, we used binary coding of items before calculating individual sum scores (0 for ‘no,’ 1 for ‘yes, once or twice,’ or for ‘yes, more than two times’). The model fit is worse than all other models, indicating the inappropriateness of comparing the age groups on simple sum scores.

**TABLE 3:** Table for 2PL model fits per MI level.

	M2	df	*p*	RMSEA	CFI	AIC	BIC
Configural MI	81.40	40	< 0.001	0.031	0.963	7909.53	7967.28

	∆M2	∆df	*p*	∆RMSEA	∆CFI	AIC	BIC
**Weak MI**	**13.44**	**7**	**0.08**	**−0.0003**	**−0.0057**	**7908.34**	**7953.46**
Strong MI	47.69	14	< 0.001	0.005	−0.0298	7931.30	7963.79
MI of sum score	172.3	7	< 0.001	0.028	−0.173	8089.58	8144.37

*Note:* Bold is the model with the best model fit characteristics.

Abbreviations: AIC, akaike information criterion; BIC, Bayesian information criterion; CFI, comparative fit index; MI, measurement invariance; RMSEA, root mean square error of approximation; SRMSR, standardised root mean square residual; TLI, Tucker‐Lewis index.

**TABLE 4:** Parameters of the weak measurement invariance model, (*β*: Item difficulty/severity, *α*: Item discrimination coefficient) (8 items, *n* = 1076 respondents).

#	Item	< 18 years (*n* = 291)		18–28 years (*n* = 785)	
		*β*	*α*	*β*	*α*
1	Been kept awake at night due to noise from drunken persons	−1.13	0.48	−0.39	0.48
2	Harassed you on the street or in a public place	−0.38	3.31	0.89	3.31
3	Yelled at you or insulted you in other ways	−1.48	2.34	−0.31	2.34
4	Has made you afraid on the street or other public place	−0.77	0.88	−0.93	0.88
5	Has insulted you at a party or other private event	−1.38	1.75	−1.34	1.75
6	Ruined clothes or belongings	−2.37	1.47	−2.23	1.47
7	Has physically harmed you	−3.45	1.45	−2.90	1.45
8	Was responsible for an auto accident in which you were involved	−4.81	1.08	−4.70	1.08
	Mean (SD)	0 (1.00)		0 (0.85)	

Table 4 summarises parameter estimates for both groups using the weak MI model. As defined by the model, discrimination values α are different between items but are the same in both groups. When examining item difficulty levels across age groups, the first four items (harassment on the street, physical harm, being yelled at, and sleep disruption) demonstrate lower severity thresholds amongst older respondents. This pattern suggests that older adults are more likely to endorse these experiences at lower levels of the underlying trait compared to younger respondents. In other words, these particular adverse experiences appear to be more readily reported or recognised by older individuals, indicating potential age‐related differences in either exposure patterns or reporting thresholds for these specific types of incidents. When researchers want to compare average scores between groups, they need the strong measurement model to make valid conclusions. The weak model isn't useful for this purpose because it automatically sets the group difference to zero instead of actually measuring it (see Table 4). Only the strong model allows researchers to directly compare group averages without making extra assumptions about the data. However, in situations where not all items work the same way across groups, researchers can still make meaningful group comparisons using a partial MI approach. This involves identifying specific items that do function consistently across groups (called anchor items) and using these as a stable reference point. By anchoring the comparison on these reliable items, researchers can still draw valid conclusions about group differences even when some other items in the test behave differently across groups. E.g., if we assume strong partial MI, for item 2 (‘Has physically harmed you’), thus making it the anchor item, a standardised group mean difference of *d* = −0.43 for the total scale difference between groups results with lower values for adults. This difference is substantially higher than the Cohen's d we get when comparing sum scores without any modelling (*d* = −0.25), emphasising the benefit of such modelling. We have not only tested implicit assumptions underlying score comparisons but also adjusted for measurement error by means of the latent variable model. The model eliminated error variance from the scores and allowed us to see the group difference clearer (as the variance is in the denominator of Cohen's d's formula). Further instructions on how to deal with violated measurement invariance can be found in [18].

### 3.6 Finding Distinct New Subgroups: IRT Trees

MI/DIF can also be approached in an exploratory fashion: for example, age groups do not have to be predefined but instead an algorithm can determine cut‐points from the data at hand. These cut‐points represent the maximised group difference in item parameters that could be found. One such exploratory technique is *regression trees*, which are applied to IRT models via the package psychotree. Additionally, to DIF analysis as described in the last section, trees can look for differences amongst several covariates. The first split will be made for the most striking group difference, the second for the second most prominent, and so on.

**FIGURE 3:**
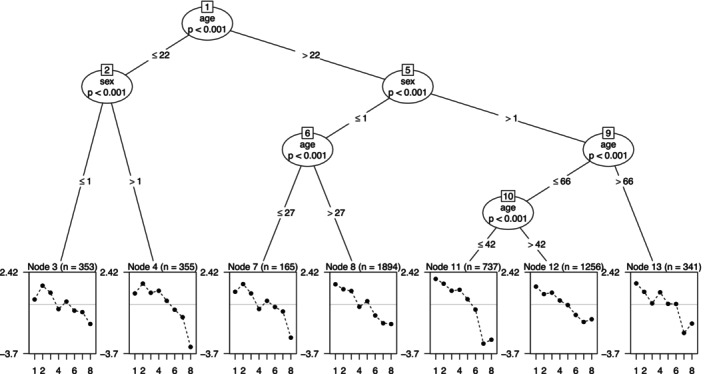
Tree with nodes for age and sex, and with items sorted by overall difficulty (8 items, *n* = 5133 respondents).

Next, we will apply this package with a polytomous Rasch model to our data and will search for differences in item parameters for gender and age (R/Stata Code in supplement). The result is an inverted tree‐like structure indicating the breaking points for a rather conservative low alpha level of 0.1%, determined by the very large sample size (Figure 3).

The most prominent difference is found for cut‐point at age 22. After this, the branches divide according to gender. The ‘older branch’ (> 22) is then further split for the ages of 27, 66, and 42. Note that the last panel to the right gives the item difficulties for females above 66, whilst the second‐to‐last right‐hand panel displays females between 42 and 66. The most striking differences were found in items 2 and 8 (2: ‘*Has physically harmed you*’; 8: ‘*Was responsible for an auto accident in which you were involved*’). Item 2 is rarely endorsed for women aged 22 to 42 or women above 66 years of age. Item 8 portrays a relatively low value in females below 22 years, 27 to 42 years, and above 66 years. The same was found for men between 22 and 27. These combinations provide unique item difficulty profiles across gender and age groups. Finding these differences in exploratory analyses is not surprising given the results of the previous MI testing. After MI tests are run, IRT trees can thus provide detailed insight into those subgroups with the largest differences in item properties.

## 4 Discussion

Simple sum scores for aggregating questionnaire responses rely on the stringent assumptions of equal item weighting and measurement invariance. These assumptions are often untenable in practise. In our example from alcohol research (8 items on alcohol harms due to others' drinking behaviour, AHTO), we have demonstrated that the assumptions of a simple sum score did not hold and how conclusions about group differences were consequently biased: Differences in AHTO between adolescents and (young) adults were substantially underestimated by the sum score (*d* = 0.25) compared to IRT modelling (*d* = 0.43).

## 5 Conclusions

Overall, IRT modelling offers a more flexible approach by explicitly testing assumptions and providing item calibrations based on realistic psychometric properties. We have demonstrated in this tutorial how to apply IRT modelling to the scaling of questionnaire items that represent a latent trait of a respondent. By using fit indices, it is easy to evaluate whether a simple Rasch model or a more complex model with relaxed assumptions (such as the 2 PL model) provides a better model fit. The assessment of different item functioning (DIF) in subgroups, meaning different item severity/weights, is one essential step when using IRT models. IRT trees might help to evaluate subgroups for which items of the questionnaire have different weights regarding the latent trait. We illustrated these approaches using actual AHTO data from the Danish general population. The provided R and Stata code (supplement) may help the reader to transfer these approaches to one's own data.

## Author Contributions

U.G., D.S., and K.B. contributed to the concept and design of this work. K.B. was responsible for data acquisition, interpretation of results, and revising the paper. D.S. conducted the analyses, provided the first draft, interpreted the results, and revised the paper. P.G. conducted parts of the analyses, interpreted the results, and revised the paper. U.G. provided parts of the first draft, interpreted the results, and revised the paper. All authors gave final approval of the version to be published. Each author certifies that their contribution to this work meets the standards of the International Committee of Medical Journal Editors.

## Disclosure

The Danish study data come from the 2011 Danish national alcohol and drug survey conducted by Statistics Denmark for the Centre for Alcohol and Drug Research.

## Conflicts of Interest

The authors declare no conflicts of interest.

## Supporting information


**Data S1:** Supporting Information.

## Data Availability

The data that support the findings of this study are openly available in Zenodo at https://zenodo.org/records/15615570, reference number 10.5281/zenodo.15615569.

## Endnotes

^1^
Note, that factor analysis is closely related to 2PL model, but originally comes from a background where items are measured on a continuous and not categorical scale.

^2^
A generalisation of the 2PL model for more than two response categories is implemented as itemtype = ‘gpcm’, which combines the features of the polytomous Rasch model and the 2PL model.

^3^
In this statement, item discriminations are called slopes and difficulties are called intercepts. Mathematically, restricting slopes comes with freely estimating the latent variance, which is why ‘slopes’ and ‘free_var’ are mentioned together. The same applies to intercepts and latent means.
