# Supplementary material for: Using Item Response Theory Models in Scaling Severity Scores in Alcohol Research—A Tutorial
Source: Drug Alcohol Rev. 2025 Oct 26;45(1):e70053. doi: 10.1111/dar.70053 (PMC12679898; doi:10.1111/dar.70053)
Supplement: Supplementary file 1 — Data S1: Supporting Information. [file DAR-45-0-s001.docx]

Supplemental Material for the Paper: Using Item Response Theory models in scaling severity scores in alcohol research - a tutorial

Short running title: A tutorial for using Item Response Theory modeling

Daniel Schulze, Kim Bloomfield, Pimrapat Gebert & Ulrike Grittner

**Content**

[1 R Session Info 3](#_Toc200208233)

[2 Complete R code for IRT Modeling (output in blue) 3](#_Toc200208234)

[### IRT modeling for IRT tutorial paper 3](#_Toc200208235)

[# data preparation 3](#_Toc200208236)

[### Rasch model 4](#_Toc200208238)

[### Partial Credit Model 5](#_Toc200208239)

[### 2PL Model 5](#_Toc200208240)

[### Measurement Invariance Analysis 6](#_Toc200208241)

[### IRT trees 7](#_Toc200208242)

[3 Complete Stata Code and output for IRT modeling 8](#_Toc200208243)

[// Data preparation 8](#_Toc200208244)

[// Rasch model-1PL model for binary items 8](#_Toc200208245)

[// Partial credit model for three responses 10](#_Toc200208246)

[// 2PL model for binary items 13](#_Toc200208247)

1 R Session Info

=== QUICK SESSION SUMMARY ===

R: R version 4.4.0 (2024-04-24 ucrt)

RStudio: 2024.4.0.735

Packages: 15 attached, 74 loaded via namespace

Date: 2025-05-29

Attached packages:

psychotree 0.16-1

psychotools 0.7-4

partykit 1.2-21

mvtnorm 1.2-5

libcoin 1.0-10

ltm 1.2-0

polycor 0.8-1

msm 1.7.1

MASS 7.3-60.2

tikzDevice 0.12.6

ggplot2 3.5.1

xtable 1.8-4

foreign 0.8-87

mirt 1.42

lattice 0.22-6

2 Complete R code for IRT Modeling (output in blue)

### IRT modeling for IRT tutorial paper

##load packages

library(mirt)

library(foreign)

library(xtable)

library(ggplot2)

library(tikzDevice)

library(ltm)

library(psychotree)

##load dataset

Dat <- read.spss("denmark_2011_2.sav", to.data.frame = T, use.value.labels = F)

# data preparation

DatHarms <- Dat[, c("item_1", "item_2","item_3","item_4","item_5", "item_6","item_7","item_8" )]

DatHarms[DatHarms == 8] <- NA # "do not want to say" is set to missing

DatHarms[DatHarms == 9] <- NA # "do not know" is set to missing

Dat2 <- Dat[complete.cases(DatHarms), ]

DatHarms <- DatHarms[complete.cases(DatHarms), ]

DatHarmsDich <- DatHarms

DatHarmsDich[DatHarmsDich < 3] <- 1 # 1 for "yes, once" or "yes, more than once"

DatHarmsDich[DatHarmsDich == 3] <- 0 # 0 for "no"

DatHarmsDich2<-DatHarmsDich

#preparation for measurement invariance analysis

age <- NULL

age[Dat2$age <= 28] <- "adult"#19-28 years old

age[Dat2$age < 18] <- "adolescence"#15-18years old

age <- age[complete.cases(age)]

#preparation for IRT trees (koen=sex, alder=age)

DatTreePC <- list()

DatTreePC$items <- as.matrix(DatHarms - 1) # rescale 3,2,1 to 2,1,0

DatTreePC$sex <- Dat2$sex

DatTreePC$age <- Dat2$age

### Rasch model

mod1<- mirt(DatHarmsDich2,model = 1,itemtype = "Rasch")

M2(mod1)

M2 df p RMSEA RMSEA_5 RMSEA_95 SRMSR TLI CFI

stats 689.7012 27 0 0.06937323 0.06494103 0.07389204 0.07965513 0.904237 0.9076571

coef(mod1, simplify = T, IRTpars=T)

$items

a b g u

item_1 1 1.709 0 1

item_2 1 1.731 0 1

item_3 1 2.121 0 1

item_4 1 2.754 0 1

item_5 1 2.853 0 1

item_6 1 4.312 0 1

item_7 1 5.096 0 1

item_8 1 6.502 0 1

$means

F1

0

$cov

F1

F1 3.265

#Figure 1: plot of Rasch model

plot(mod1, type = 'trace', facet_items=FALSE, theta_lim = c(-5, 12), strip = FALSE, par.settings = list(superpose.line = list(col = c("black", "firebrick2", "dodgerblue",'seagreen2', "dodgerblue", "firebrick2", "black",'seagreen2'),lty = c("solid", "dashed","solid", "dashed", "dashed", "solid", "dashed", "solid"), lwd=2)), auto.key= list(text = c("Item 1","Item 2", "Item 3", "Item 4", "Item 5", "Item 6", "Item 7", "Item 8"), space="right", columns=1, title="Legend", cex.title=1, lines=TRUE, points=FALSE))


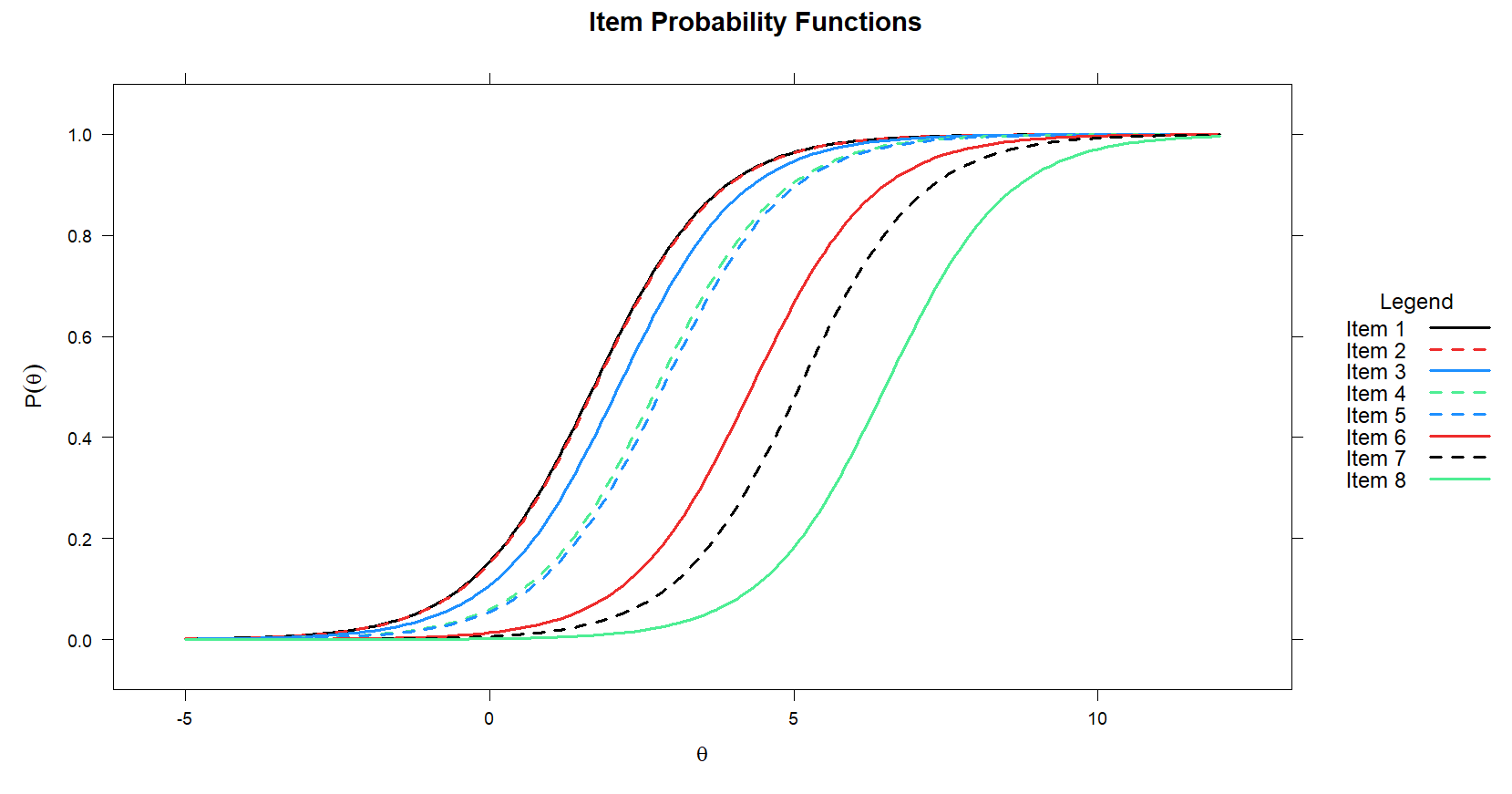


### Partial Credit Model

mod2 <- mirt(DatHarms, model = 1, itemtype = "Rasch")

M2(mod2)

M2 df p RMSEA RMSEA_5 RMSEA_95 SRMSR TLI CFI

stats 196.903 19 0 0.04284792 0.03753446 0.04836697 0.09340082 0.9505952 0.9530655

coef(mod2, simplify = T)

$items

a1 ak0 ak1 ak2 d0 d1 d2

item_1 1 0 1 2 0 2.464 4.287

item_2 1 0 1 2 0 2.567 4.392

item_3 1 0 1 2 0 2.922 5.074

item_4 1 0 1 2 0 3.483 6.175

item_5 1 0 1 2 0 3.730 6.489

item_6 1 0 1 2 0 3.839 8.016

item_7 1 0 1 2 0 3.983 8.917

item_8 1 0 1 2 0 4.063 10.399

$means

F1

0

$cov

F1

F1 2.382

### 2PL Model

mod3 <- mirt(DatHarmsDich2, model = 1, itemtype = "2PL")

M2(mod3)

M2 df p RMSEA RMSEA_5 RMSEA_95 SRMSR TLI CFI

stats 126.667 20 0 0.03233814 0.02707988 0.03783293 0.03471006 0.9791913 0.9851367

coef(mod3, simplify = T)

$items

a1 d g u

item_1 0.844 -1.267 0 1

item_2 3.744 -2.908 0 1

item_3 2.766 -2.793 0 1

item_4 1.523 -2.522 0 1

item_5 1.880 -2.909 0 1

item_6 2.097 -4.661 0 1

item_7 2.228 -5.682 0 1

item_8 1.268 -5.791 0 1

$means

F1

0

$cov

F1

F1 1

#Figure 2: Plot of 2PL model

plot(mod3, type = 'trace', facet_items=FALSE, theta_lim = c(-5, 12), strip = FALSE, par.settings = list(superpose.line = list(col = c("black", "firebrick2", "dodgerblue",'seagreen2', "dodgerblue", "firebrick2", "black",'seagreen2'), lty = c("solid", "dashed","solid", "dashed", "dashed", "solid", "dashed", "solid"), lwd=2)), auto.key= list(text = c("Item 1","Item 2", "Item 3", "Item 4", "Item 5", "Item 6", "Item 7", "Item 8"), space="right", columns=1, title="Legend", cex.title=1, lines=TRUE, points=FALSE))


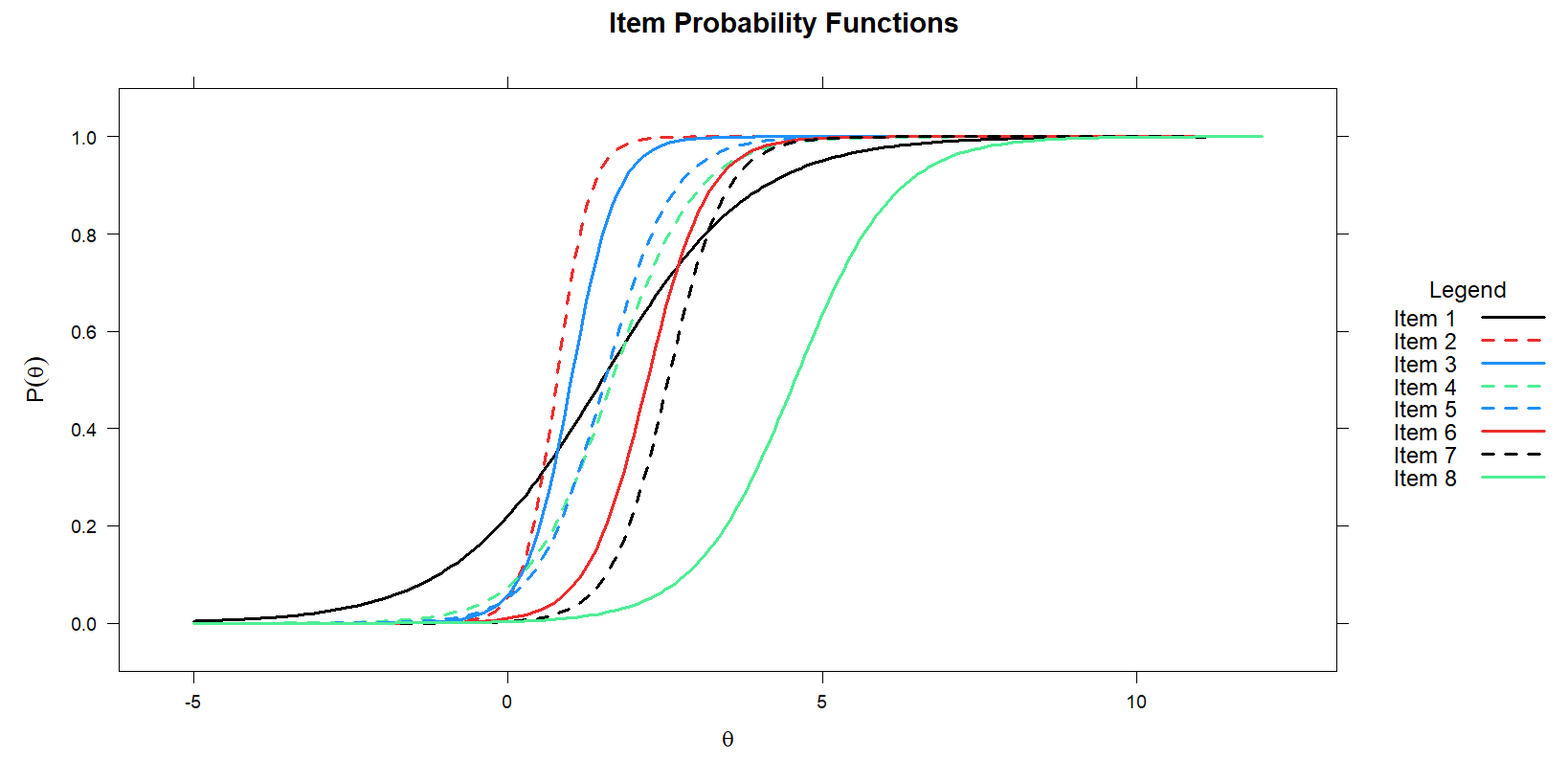


### Measurement Invariance Analysis

modMIconfigural <- multipleGroup(DatHarmsDich[Dat2$age <=28, ], model = 1, itemtype = "2PL", group = age)

modMIweak<- multipleGroup(DatHarmsDich[Dat2$age <=28, ], model = 1, itemtype = "2PL", group = age, invariance = c("slopes", "free_var"))

modMIstrong <- multipleGroup(DatHarmsDich[Dat2$age <=28, ], model = 1, itemtype = "2PL", group = age, SE=T, invariance = c("slopes", "intercepts", "free_mean", "free_var"))

anova(modMIstrong, modMIweak)

anova(modMIweak, modMIconfigural)

#model parameter for table 4

coef(modMIweak, simplify=T)

$adolescence

$items

a1 d g u

item_1 0.479 -1.130 0 1

item_2 3.307 -0.377 0 1

item_3 2.343 -1.479 0 1

item_4 0.882 -0.774 0 1

item_5 1.747 -1.378 0 1

item_6 1.469 -2.374 0 1

item_7 1.449 -3.452 0 1

item_8 1.079 -4.812 0 1

$means

F1

0

$cov

F1

F1 1

$adult

$items

a1 d g u

item_1 0.479 -0.385 0 1

item_2 3.307 0.885 0 1

item_3 2.343 -0.313 0 1

item_4 0.882 -0.933 0 1

item_5 1.747 -1.335 0 1

item_6 1.469 -2.226 0 1

item_7 1.449 -2.904 0 1

item_8 1.079 -4.704 0 1

$means

F1

0

$cov

F1

F1 0.824

### IRT trees

modTreePCRestr <- pctree(items ~ sex + age, data = DatTreePC, alpha = .001, nullcats = "downcode")

plot(modTreePCRestr, type = "profile")


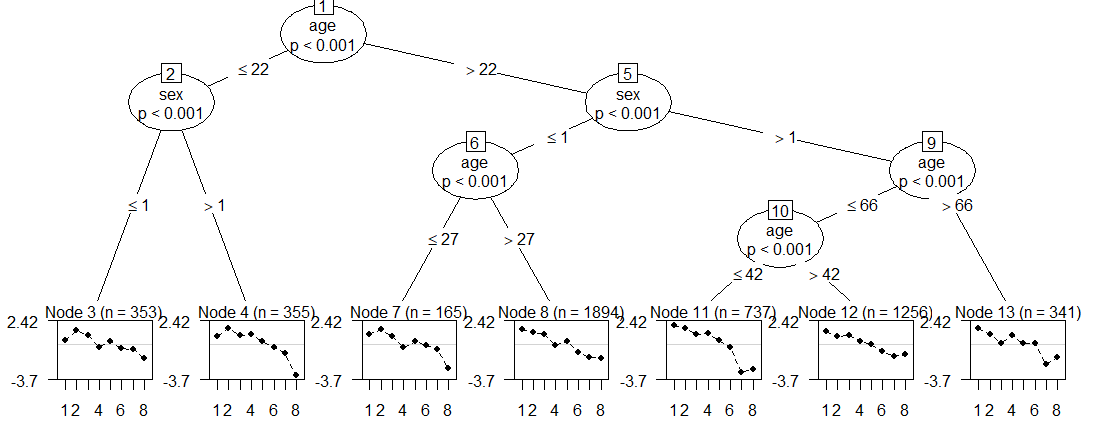


3 Complete Stata Code and output for IRT modeling

***Stata/MP 18.0**

## **// Data preparation**

use "denmark_2011_2.dta", clear

//recode

foreach x of varlist item_2-item_8 {

replace `x'=. if `x'==8 | `x'==9 //recode to missing

drop if `x'==. //delete all the missing

}

count //keep only complete case n=5,101

//example to explore the code and data

. codebook item_1

------------------------------------------------------------------------------------------------

item_1

------------------------------------------------------------------------------------------------

Type: Numeric (double)

Label: item_1

Range: [1,3] Units: 1

Unique values: 3 Missing .: 0/5,101

Tabulation: Freq. Numeric Label

389 1 yes, more than 2 times

880 2 yes, once or twice

3,832 3 no

*Recode data -> binary

foreach x of varlist item_1-item_8 {

gen `x'_yn=`x'<3

}

label define yn 0"No" 1"Yes"

label value item_1_yn-item_8_yn yn

## **// Rasch model-1PL model for binary items**

. gsem (B -> (item_1_yn-item_8_yn)@1), logit nodvheader var(B@3.265)

Fitting fixed-effects model:

Iteration 0: Log likelihood = -14595.168

Iteration 1: Log likelihood = -13998.525

Iteration 2: Log likelihood = -13962.343

Iteration 3: Log likelihood = -13961.303

Iteration 4: Log likelihood = -13961.301

Iteration 5: Log likelihood = -13961.301

Refining starting values:

Grid node 0: Log likelihood = -12726.84

Fitting full model:

Iteration 0: Log likelihood = -12726.84

Iteration 1: Log likelihood = -12360.19

Iteration 2: Log likelihood = -12358.48

Iteration 3: Log likelihood = -12358.479

Iteration 4: Log likelihood = -12358.479

Generalized structural equation model Number of obs = 5,101

Log likelihood = -12358.479

( 1) [item_1_yn]B = 1

( 2) [item_2_yn]B = 1

( 3) [item_3_yn]B = 1

( 4) [item_4_yn]B = 1

( 5) [item_5_yn]B = 1

( 6) [item_6_yn]B = 1

( 7) [item_7_yn]B = 1

( 8) [item_8_yn]B = 1

( 9) [/]var(B) = 3.265

------------------------------------------------------------------------------

| Coefficient Std. err. z P>|z| [95% conf. interval]

-------------+----------------------------------------------------------------

item_1_yn |

B | 1 (constrained)

_cons | -1.708857 .0475958 -35.90 0.000 -1.802143 -1.615571

-------------+----------------------------------------------------------------

item_2_yn |

B | 1 (constrained)

_cons | -1.730682 .0477113 -36.27 0.000 -1.824194 -1.637169

-------------+----------------------------------------------------------------

item_3_yn |

B | 1 (constrained)

_cons | -2.120052 .0500827 -42.33 0.000 -2.218212 -2.021892

-------------+----------------------------------------------------------------

item_4_yn |

B | 1 (constrained)

_cons | -2.752666 .0553687 -49.72 0.000 -2.861186 -2.644145

-------------+----------------------------------------------------------------

item_5_yn |

B | 1 (constrained)

_cons | -2.852201 .056392 -50.58 0.000 -2.962727 -2.741674

-------------+----------------------------------------------------------------

item_6_yn |

B | 1 (constrained)

_cons | -4.312319 .080419 -53.62 0.000 -4.469937 -4.1547

-------------+----------------------------------------------------------------

item_7_yn |

B | 1 (constrained)

_cons | -5.098676 .1042121 -48.93 0.000 -5.302928 -4.894424

-------------+----------------------------------------------------------------

item_8_yn |

B | 1 (constrained)

_cons | -6.512659 .1834179 -35.51 0.000 -6.872152 -6.153167

-------------+----------------------------------------------------------------

var(B)| 3.265 (constrained)

------------------------------------------------------------------------------

**Alternative command**

. irt 1pl item_1_yn -item_8_yn, estmetric

. gsem (B -> (item_1_yn -item_8_yn)@1), logit nodvheader

**Note:** the results from irt 1pl provide a slightly different number regarding the variance.

## **// Partial credit model for three responses**

. irt pcm item_1-item_8

Fitting fixed-effects model:

Iteration 0: Log likelihood = -16888.201

Iteration 1: Log likelihood = -16888.201

Fitting full model:

Iteration 0: Log likelihood = -15799.39

Iteration 1: Log likelihood = -15176.81

Iteration 2: Log likelihood = -15102.214

Iteration 3: Log likelihood = -15101.685

Iteration 4: Log likelihood = -15101.687

Iteration 5: Log likelihood = -15101.687

Partial credit model Number of obs = 5,101

Log likelihood = -15101.687

------------------------------------------------------------------------------

| Coefficient Std. err. z P>|z| [95% conf. interval]

-------------+----------------------------------------------------------------

Discrim | 1.563444 .0372739 41.94 0.000 1.490388 1.636499

-------------+----------------------------------------------------------------

item_1 |

Diff |

2 vs 1 | -1.584238 .0481375 -32.91 0.000 -1.678585 -1.48989

3 vs 2 | -1.168104 .0354825 -32.92 0.000 -1.237648 -1.098559

-------------+----------------------------------------------------------------

item_2 |

Diff |

2 vs 1 | -1.650198 .0493602 -33.43 0.000 -1.746942 -1.553454

3 vs 2 | -1.169537 .0353431 -33.09 0.000 -1.238808 -1.100265

-------------+----------------------------------------------------------------

item_3 |

Diff |

2 vs 1 | -1.878616 .0568389 -33.05 0.000 -1.990018 -1.767213

3 vs 2 | -1.37908 .0381944 -36.11 0.000 -1.45394 -1.30422

-------------+----------------------------------------------------------------

item_4 |

Diff |

2 vs 1 | -2.23921 .0734057 -30.50 0.000 -2.383082 -2.095337

3 vs 2 | -1.725758 .0441491 -39.09 0.000 -1.812288 -1.639227

-------------+----------------------------------------------------------------

item_5 |

Diff |

2 vs 1 | -2.397313 .0803128 -29.85 0.000 -2.554723 -2.239903

3 vs 2 | -1.768766 .0448397 -39.45 0.000 -1.85665 -1.680882

-------------+----------------------------------------------------------------

item_6 |

Diff |

2 vs 1 | -2.469943 .1187948 -20.79 0.000 -2.702776 -2.237109

3 vs 2 | -2.677321 .0699106 -38.30 0.000 -2.814343 -2.540299

-------------+----------------------------------------------------------------

item_7 |

Diff |

2 vs 1 | -2.563368 .1603426 -15.99 0.000 -2.877634 -2.249102

3 vs 2 | -3.162902 .0896827 -35.27 0.000 -3.338677 -2.987127

-------------+----------------------------------------------------------------

item_8 |

Diff |

2 vs 1 | -2.618836 .2862635 -9.15 0.000 -3.179902 -2.05777

3 vs 2 | -4.05987 .1492042 -27.21 0.000 -4.352305 -3.767435

------------------------------------------------------------------------------

For item item_1, a person with the difficulties -1.58 is equally likely to answer “yes, once or twice or yes, more than two times” (labeled 2 vs 1). A person with -1.17 is equally likely to answer “No or yes, once or twice” (labeled 3 vs 2).

To get the difficulty parameters per item in Stata, we need to use option estmetric after the model. For item_1, β_1_ = 2.48 for crossing over from “yes, more than two times” to “yes, once or twice”, and β_2_ = 4.30 for changing from the latter to “no”.

. irt pcm item_1-item_8, estmetric

Fitting fixed-effects model:

Iteration 0: Log likelihood = -16888.201

Iteration 1: Log likelihood = -16888.201

Fitting full model:

Iteration 0: Log likelihood = -15799.39

Iteration 1: Log likelihood = -15176.81

Iteration 2: Log likelihood = -15102.214

Iteration 3: Log likelihood = -15101.685

Iteration 4: Log likelihood = -15101.687

Iteration 5: Log likelihood = -15101.687

Partial credit model Number of obs = 5,101

Log likelihood = -15101.687

( 1) [2.item_1]Theta - [2.item_8]Theta = 0

( 2) [3.item_1]Theta - 2*[2.item_8]Theta = 0

( 3) [2.item_2]Theta - [2.item_8]Theta = 0

( 4) [3.item_2]Theta - 2*[2.item_8]Theta = 0

( 5) [2.item_3]Theta - [2.item_8]Theta = 0

( 6) [3.item_3]Theta - 2*[2.item_8]Theta = 0

( 7) [2.item_4]Theta - [2.item_8]Theta = 0

( 8) [3.item_4]Theta - 2*[2.item_8]Theta = 0

( 9) [2.item_5]Theta - [2.item_8]Theta = 0

(10) [3.item_5]Theta - 2*[2.item_8]Theta = 0

(11) [2.item_6]Theta - [2.item_8]Theta = 0

(12) [3.item_6]Theta - 2*[2.item_8]Theta = 0

(13) [2.item_7]Theta - [2.item_8]Theta = 0

(14) [3.item_7]Theta - 2*[2.item_8]Theta = 0

(15) - 2*[2.item_8]Theta + [3.item_8]Theta = 0

(16) [/]var(Theta) = 1

------------------------------------------------------------------------------

| Coefficient Std. err. z P>|z| [95% conf. interval]

-------------+----------------------------------------------------------------

1.item_1 | (base outcome)

-------------+----------------------------------------------------------------

2.item_1 |

Theta | 1.563444 .0372739 41.94 0.000 1.490388 1.636499

_cons | 2.476866 .0822139 30.13 0.000 2.31573 2.638002

-------------+----------------------------------------------------------------

3.item_1 |

Theta | 3.126887 .0745478 41.94 0.000 2.980776 3.272998

_cons | 4.30313 .0990255 43.45 0.000 4.109044 4.497217

-------------+----------------------------------------------------------------

1.item_2 | (base outcome)

-------------+----------------------------------------------------------------

2.item_2 |

Theta | 1.563444 .0372739 41.94 0.000 1.490388 1.636499

_cons | 2.579991 .0835629 30.87 0.000 2.416211 2.743772

-------------+----------------------------------------------------------------

3.item_2 |

Theta | 3.126887 .0745478 41.94 0.000 2.980776 3.272998

_cons | 4.408496 .1004831 43.87 0.000 4.211552 4.605439

-------------+----------------------------------------------------------------

1.item_3 | (base outcome)

-------------+----------------------------------------------------------------

2.item_3 |

Theta | 1.563444 .0372739 41.94 0.000 1.490388 1.636499

_cons | 2.937109 .0936863 31.35 0.000 2.753487 3.120731

-------------+----------------------------------------------------------------

3.item_3 |

Theta | 3.126887 .0745478 41.94 0.000 2.980776 3.272998

_cons | 5.093223 .11082 45.96 0.000 4.87602 5.310426

-------------+----------------------------------------------------------------

1.item_4 | (base outcome)

-------------+----------------------------------------------------------------

2.item_4 |

Theta | 1.563444 .0372739 41.94 0.000 1.490388 1.636499

_cons | 3.500878 .1168013 29.97 0.000 3.271951 3.729804

-------------+----------------------------------------------------------------

3.item_4 |

Theta | 3.126887 .0745478 41.94 0.000 2.980776 3.272998

_cons | 6.199002 .1329626 46.62 0.000 5.938401 6.459604

-------------+----------------------------------------------------------------

1.item_5 | (base outcome)

-------------+----------------------------------------------------------------

2.item_5 |

Theta | 1.563444 .0372739 41.94 0.000 1.490388 1.636499

_cons | 3.748064 .1260109 29.74 0.000 3.501087 3.99504

-------------+----------------------------------------------------------------

3.item_5 |

Theta | 3.126887 .0745478 41.94 0.000 2.980776 3.272998

_cons | 6.513429 .1416784 45.97 0.000 6.235745 6.791114

-------------+----------------------------------------------------------------

1.item_6 | (base outcome)

-------------+----------------------------------------------------------------

2.item_6 |

Theta | 1.563444 .0372739 41.94 0.000 1.490388 1.636499

_cons | 3.861616 .1880029 20.54 0.000 3.493137 4.230095

-------------+----------------------------------------------------------------

3.item_6 |

Theta | 3.126887 .0745478 41.94 0.000 2.980776 3.272998

_cons | 8.047456 .1954293 41.18 0.000 7.664422 8.43049

-------------+----------------------------------------------------------------

1.item_7 | (base outcome)

-------------+----------------------------------------------------------------

2.item_7 |

Theta | 1.563444 .0372739 41.94 0.000 1.490388 1.636499

_cons | 4.007681 .2529545 15.84 0.000 3.511899 4.503462

-------------+----------------------------------------------------------------

3.item_7 |

Theta | 3.126887 .0745478 41.94 0.000 2.980776 3.272998

_cons | 8.952699 .2515349 35.59 0.000 8.4597 9.445699

-------------+----------------------------------------------------------------

1.item_8 | (base outcome)

-------------+----------------------------------------------------------------

2.item_8 |

Theta | 1.563444 .0372739 41.94 0.000 1.490388 1.636499

_cons | 4.094402 .4498287 9.10 0.000 3.212754 4.97605

-------------+----------------------------------------------------------------

3.item_8 |

Theta | 3.126887 .0745478 41.94 0.000 2.980776 3.272998

_cons | 10.44178 .4203282 24.84 0.000 9.617951 11.26561

-------------+----------------------------------------------------------------

var(Theta)| 1 (constrained)

------------------------------------------------------------------------------

## **// 2PL model for binary items**

. irt 2pl item_1_yn -item_8_yn, estmetric intpoints(21)

Fitting fixed-effects model:

Iteration 0: Log likelihood = -14595.168

Iteration 1: Log likelihood = -13998.525

Iteration 2: Log likelihood = -13962.343

Iteration 3: Log likelihood = -13961.303

Iteration 4: Log likelihood = -13961.301

Iteration 5: Log likelihood = -13961.301

Fitting full model:

Iteration 0: Log likelihood = -13783.538 (not concave)

Iteration 1: Log likelihood = -12476.056

Iteration 2: Log likelihood = -12444.126

Iteration 3: Log likelihood = -12350.209 (backed up)

Iteration 4: Log likelihood = -12123.207

Iteration 5: Log likelihood = -12114.484

Iteration 6: Log likelihood = -12114.341

Iteration 7: Log likelihood = -12114.341

Two-parameter logistic model Number of obs = 5,101

Log likelihood = -12114.341

( 1) [/]var(Theta) = 1

------------------------------------------------------------------------------

| Coefficient Std. err. z P>|z| [95% conf. interval]

-------------+----------------------------------------------------------------

item_1_yn |

Theta | .8445432 .0500439 16.88 0.000 .7464591 .9426274

_cons | -1.267057 .040269 -31.46 0.000 -1.345983 -1.188131

-------------+----------------------------------------------------------------

item_2_yn |

Theta | 3.762646 .2670836 14.09 0.000 3.239172 4.28612

_cons | -2.917253 .186895 -15.61 0.000 -3.283561 -2.550946

-------------+----------------------------------------------------------------

item_3_yn |

Theta | 2.764856 .1470277 18.81 0.000 2.476687 3.053025

_cons | -2.790177 .1245761 -22.40 0.000 -3.034341 -2.546012

-------------+----------------------------------------------------------------

item_4_yn |

Theta | 1.52411 .0789211 19.31 0.000 1.369428 1.678793

_cons | -2.521786 .0775471 -32.52 0.000 -2.673776 -2.369797

-------------+----------------------------------------------------------------

item_5_yn |

Theta | 1.879397 .0974261 19.29 0.000 1.688445 2.070348

_cons | -2.90769 .0992752 -29.29 0.000 -3.102265 -2.713114

-------------+----------------------------------------------------------------

item_6_yn |

Theta | 2.095431 .1460923 14.34 0.000 1.809095 2.381766

_cons | -4.65737 .1979224 -23.53 0.000 -5.04529 -4.269449

-------------+----------------------------------------------------------------

item_7_yn |

Theta | 2.2255 .1938543 11.48 0.000 1.845553 2.605447

_cons | -5.675977 .2974281 -19.08 0.000 -6.258925 -5.093029

-------------+----------------------------------------------------------------

item_8_yn |

Theta | 1.266289 .2242079 5.65 0.000 .8268502 1.705729

_cons | -5.787757 .3109142 -18.62 0.000 -6.397138 -5.178376

-------------+----------------------------------------------------------------

var(Theta)| 1 (constrained)

------------------------------------------------------------------------------

From the output, we used option estmetric for displaying the item discrimination parameters (Theta = 𝛼) and _cons = β. The intpoints(21) is set because it is the default setting in mirt, but Stata sets intpoints(7)as a default.
